# Mechanical Properties of Furnace Slag and Coal Gangue Mixtures Stabilized by Cement and Fly Ash

**DOI:** 10.3390/ma14227103

**Published:** 2021-11-22

**Authors:** Hongbo Li, Hubiao Zhang, Pengfei Yan, Changyu Yan, Yufei Tong

**Affiliations:** 1 College of Civil and Hydraulic Engineering, Ningxia University, Yinchuan 750021, China; zhanghubiao1222@163.com (H.Z.); ycy7584@163.com (C.Y.); tongyufei1028@163.com (Y.T.); 2 Ningxia Research Center of Technology on Water-Saving Irrigation and Water Resources Regulation, Yinchuan 750021, China; 3 Ningxia Center of Research on Earthquake Protection and Disaster Mitigation in Civil Engineering, Yinchuan 750021, China; 4 State Key Laboratory of Deep Geotechnical Mechanics and Underground Engineering, China University of Mining and Technology, Xuzhou 221116, China; ypfgzyx@163.com

**Keywords:** no lateral limit compressive strength, split test, triaxial test, peripheral pressure

## Abstract

The mechanical properties and strength formation mechanism of cement–fly-ash-stabilized slag–coal gangue mixture were examined using an unconfined compressive strength test, splitting strength test, triaxial test, and scanning electron microscopy to solve the limitations of land occupation and environmental pollution that is caused by fly ash from the Xixia District thermal power plant in Yinchuan, slag from the Ningdong slag yard, and washed coal gangue. Its performance as a pavement base mixture on the road was investigated. The results demonstrated that as the slag replacement rate increased, the maximum water content increased while the maximum dry density decreased. The addition of slag reduced the unconfined compressive strength and splitting strength of the specimens; furthermore, the higher the slag substitution rate, the lower the unconfined compressive strength and splitting strength of the specimens. As the cement content increased, the specimen’s unconfined compressive strength increased. Based on the principle of considering the mechanical properties and economic concerns, the slag replacement rate in the actual construction should be ~50% and should not exceed 75%. Based on the relationship between the compressive strength and splitting strength of ordinary concrete, the relationship model between the unconfined compressive strength and splitting strength of cement–fly-ash-stabilized slag–coal gangue was established. The failure mode, stress–strain curve, peak stress, and failure criterion of these specimens were analyzed based on the triaxial test results, and the relationship formulas between the slag substitution rate, cement content, peak stress, and confining pressure were fitted. As per the SEM results, the mixture’s hydration products primarily included amorphous colloidal C-S-H, needle rod ettringite AFt, unhydrated cement clinker particles, and fly ash particles. The analysis of the mixture’s strength formation mechanism showed that the mixture’s strength was the comprehensive embodiment of all factors, such as the microaggregate effect, secondary hydration reaction, and material characteristics.

## 1. Introduction

Coal gangue is a rock type with lower carbon content and higher hardness compared with coal. Coal gangue comprises multiple rocks and forms one of the primary wastes in coal production. Although >300 million tons of coal gangue are discarded each year, China’s utilization rate of coal gangue has reduced [1,2,3,4]. At the moment, China has accumulated >8 billion tons of coal gangue, which covers an area of ~70 km^2^ and causes multiple environmental issues. Consequently, using coal gangue as a road base filling material can not only prevent pollution but also reduce natural resource exploitation, thus paving the approach for high-value and large-scale resource utilization [5,6,7,8].

The preparation of a coal-gangue-reinforced base mixture has been extensively investigated. Zhu et al. [9] examined the influence of polypropylene fibers on the fluidity, strength, cracking, and dry shrinkage performance of gangue mortar and reported that polypropylene fibers reduced the fluidity of gangue mortar but improved its compressive and flexural strengths. Furthermore, they reported that polypropylene fibers effectively reduced the risk of gangue mortar’s dry shrinkage and cracking. Guangyu et al. [10] prepared coal gangue through a selective crushing and separation process and examined the effects of the mixture ratio and stability type on the crushing resistance and mechanical properties of coalgangue-based materials. The results demonstrated that selective crushing obviously reduced the crushing value and calorific value of coal gangue; however, after adding 10% fly ash, it played a buffer role in the base mixture, and the unconfined compressive strength increased by 30.9% to 3.52 MPa in 7 days. Zhang et al. [11] prepared a mixture of calcareous coal gangue, fly ash, lime powder, and a small amount of cement in the Hanxing area and examined the influence. When the amount of lime powder was extremely high, the lime negatively impacted the cleavage strength of a calcareous coal gangue mixture. The beneficial effect of cement on the cracking strength of a coal gangue–cement mixture gradually decreased. Jian [12] activated fly ash and coal gangue furnace slag with chemical components and analyzed them using X-ray diffraction (XRD) and SEM. The results demonstrated that the crystal structure of the mixture changed upon calcination. Less cement hydration products were created, the structure was compact, and the early strength was high. Zhiguo et al. [13] examined the technical feasibility of applying lime–fly-ash-stabilized coal gangue to a pavement base and examined the unconfined compressive strength of the mixture under the action of water saturation. The results demonstrated that when the mass fraction of lime, fly ash, and coal gangue was 5, 15, and 80%, respectively, and the cement content was 3%, the unconfined compressive strength of the mixture met the design requirements. Navid Chalangaran et al. [14] measured the 7-, 14-, and 28-day compressive strengths of concrete by controlling the sample size and rubber replacement rate to examine the procedures and materials to improve the sound transmission loss of concrete and reduce the influence of sound transmission to residential buildings due to the mechanical properties of the materials. The results demonstrated that the sound transmission loss of samples containing 15% fine rubber powder was as high as 190%, whereas that of samples containing 15% coarse rubber powder was as high as 228%. Ji et al. [15] established a freeze–thaw damage model of coal gangue concrete using the PBS parallel-bar mechanical model; furthermore, the calculated results agreed well with the test data. Navid Chalangaran and colleagues [16] addressed the issue of excessive waste rubber tire production in transportation, where this ductile material was added to concrete to improve the toughness of the mixture; however, this rubber material reduced the strength of the concrete. Therefore, the rubber that was crushed with sizes from 1 to 3 and 3 to 6 mm was replaced by 5, 10, and 15% sand. The study reported that concrete aggregates could be proportionally replaced with crumb rubber and large amounts of discarded rubber. Compared with traditional concrete, the crumb-rubber-containing concrete had better integration; therefore, it had appropriate fluidity and durability. However, to date, existing studies on the durability and shrinkage performance of coal gangue mostly concerned contractile properties or a single mechanical property of coal gangue; therefore, a comprehensive investigation of the mechanical properties of coal gangue as a road base filling material has practical implications.

Based on the abovementioned problems, this study used cement, fly ash, slag, and coal gangue to prepare a road base mixture; performed unconfined compressive strength tests, splitting strength tests, triaxial tests, and scanning electron microscopy tests of eight types of mixtures with different proportions; and examined the variation law of mechanical properties and the strength formation mechanism of mixtures with different admixtures. Note that five types of mixtures with different slag substitution rates were tested to obtain the optimal slag substitution rate. The mechanical properties of the cement–fly-ash-stabilized slag–coal gangue mixture were comprehensively evaluated, which provided a theoretical basis for practical engineering.

## 2. Materials and Methods

### 2.1. Materials

Fly ash and furnace slag were obtained from the Xixia Thermal Power Plant in Yinchuan, Ningxia. The cement was Jockey Grade 42.5 ordinary Portland cement. Coal gangue was obtained from the local Helan Mountain in Yinchuan.

### 2.2. Methods

**Figure 1:**
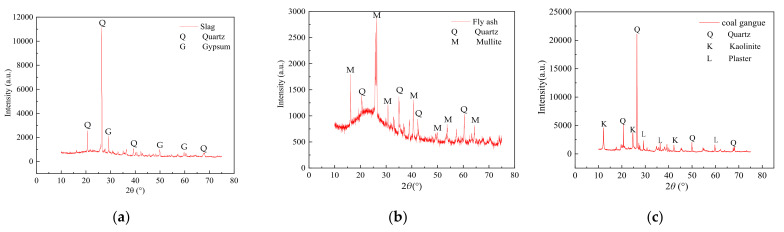
XRD patterns of furnace slag and fly ash: (**a**) furnace slag, (**b**) fly ash, and (**c**) coal gangue.

XRD and XRF were used to examine the mineralogical and chemical compositions of the slag, fly ash, and coal gangue. Figure 1 shows the XRD test results and Table 1 shows the XRF test results. The crushing value, apparent density, bulk density, and water absorption of the coal gangue with 0–4.75 mm furnace slag and four particle size grades were examined. Table 2 shows the test results.

As per Figure 1, the crystallized substances of the fly ash and furnace slag were primarily quartz, mullite, and a small amount of gypsum. Quartz is one of the primary rock-forming minerals and its primary component is SiO_2_. Mullite, also known as monalite, is a series of minerals that are composed of aluminosilicate, and its primary component is 3Al_2_O_3_·2SiO_2_. Gypsum primarily comprises sulfate minerals and its main component is CaSO_4_·nH_2_O. The primary components of the fly ash and furnace slag, as shown in Table 1, are SiO_2_, Al_2_O_3_, Fe_2_O_3_, and trace amounts of CaO and K_2_O. The SiO_2_ and Al_2_O_3_ mass fractions in the fly ash were large, accounting for ~78% of the total mass, and the burning loss was <10%, thus meeting the performance control standard of a highway coalgangue-filled sub-grade [17]. The mass fractions of SiO_2_ and Al_2_O_3_ in the furnace slag accounted for ~70% of the total mass; however, the proportion of basic oxides accounted for ~14% of the total mass. Consequently, the furnace slag was highly active and weakly alkaline. The phase composition of furnace slag is primarily a glass matrix that is made of nCaO·SiO_2_ in which basic oxides react with SiO_2_, Al_2_O_3_, and Fe_2_O_3_ after the hydration reaction, thus showing the hard cementing property of the effluent [18,19,20,21,22]. Based on the chemical reaction mechanism of the material and strength formation mechanism of the specimen, it is feasible to use furnace slag as an admixture of the pavement base.

The natural particle size distribution of the slag was uneven and very different, which needed to be broken and screened. Table 3 lists the screening results for the slag.

Table 3 shows that the slag with a particle size of <4.75 mm accounted for 76% of the total mass. On the one hand, the slag should be used in engineering as little as possible without complex technical treatment to save money and improve its engineering application value. On the other hand, the influence of the slag particle size difference on the specimen strength should be minimized as much as possible. Based on the foregoing, the slag should be passed via a 4.75 mm square hole sieve to improve the slag uniformity [23,24].

This study followed the following three principles when determining the range of the slag content. The first aim was to ensure that the gradation of the mixture after adding the slag met the requirements of cement–fly-ash-stabilized materials in the specification such that the gradation of the mixture was relatively excellent. The second aim was to keep the unconfined compressive strength of the mixture from being affected by too much fine material after adding slag. The third aim was to maximize the utilization rate of slag solid waste. Based on the foregoing considerations and the characteristics of slag and coal gangue, the 0–4.75 mm content in the slag was higher; however, the 0–4.75 mm content in the coal gangue was lower. The crushing treatment of the slag and coal gangue was minimized to the greatest extent possible. Finally, it was decided that the 0–4.75 mm coal gangue should be replaced with 0–4.75 mm slag.

**Figure 2:**
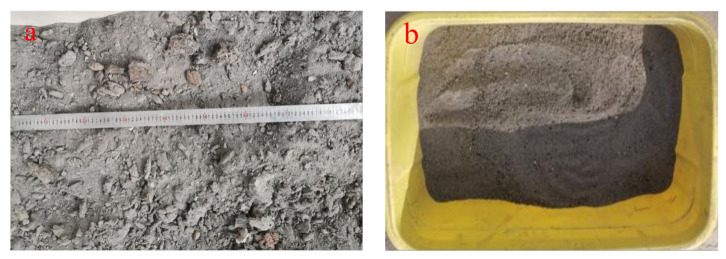
Slag before and after screening: (**a**) without screening treatment. These slag particles are poorly graded and can not be directly used in construction. Therefore, through screening test, the undisturbed slag is screened into 0–4.75 mm and the particle size range in (**b**) is experimentally studied.

As shown in Figure 2a, the natural particle size of the furnace slag was unevenly distributed and greatly varied. Mixing it directly with other materials would cause large deviations in the test results. To reduce the consumption of graded coal gangue and avoid test deviations, the furnace slag was sieved via a screen with 4.75 mm^2^ holes and replaced with 0–4.75 mm coal gangue. Figure 2b shows furnace slag after the screening. The relevant requirements of CJJ1-2008 (Code for Construction and Quality Acceptance of Urban Road Engineering [25]) divide coal gangue grading into four grades: 0–4.75, 4.75–9.5, 9.5–16, and 16–26.5 mm. Note that the fly ash was grade III ash and the fineness percentage was 17.9%. Moreover, the basic properties of the graded coal gangue and 0–4.75 mm furnace slag were evaluated; the results are shown in Table 2. Based on the existing studies [26,27], five mixtures with various furnace slag substitution rates were selected and the optimal furnace slag substitution rate was obtained; then, by considering the cement as a variable, the economic dosage of cement was found. Table 4 shows the results of the mixing ratio design.The slag before and after the screening is shown in Figure 2.

### 2.3. Test Schemes

#### 2.3.1. Compaction Testing

The compaction test is a method of overcoming the friction resistance between particles, reducing the gap between particles, and improving the compactness of the mixture. To some extent, the compaction test can show the actual construction’s compaction process, and the maximum dry density and best water content of the mixture can be obtained via the compaction test, laying the groundwork for the specimen formed using the static pressure method.

#### 2.3.2. Mechanical Property Testing

The unconfined compressive strength tests and splitting tests were performed following JTGE51-2009 (Test Specification for Stabilized Materials of Highway Engineering Inorganic Bonds [28]). The tests were performed at 7, 28, 56, and 90 days using the Shanghai Xinsansi universal testing machine. The maximum pressure of the press was 1000 kN and the loading rate was controlled at 1 mm/min.

#### 2.3.3. Triaxial Test

To show the failure mechanism of the cement–fly-ash-stabilized furnace slag and coal gangue mixtures under triaxial compression, triaxial shear strength tests were performed. A YY-RBSZ-1000 rock triaxial (creep) tester developed by Rugao Yuanye Survey Machinery Factory was adopted [29].

The unit features a triaxial pressure unit, two hydraulic pumps, displacement sensors, and pressure sensors, with one hydraulic pump to provide vertical load and one hydraulic pump to provide a stable confining pressure. The maximum axial pressure is 1000 kN, the maximum confining pressure is 30 MPa, the static accuracy of axial and confining pressure loading is 0.5%, and the temperature stability of the axial and confining pressure loading is 0.5%/°C.

#### 2.3.4. SEM Test

Scanning electron microscopy was undertaken using an EVO 18 tungsten filament scanning electron microscope produced by ZEISS (Oberkochen, Germany) which has a resolution of 3 nm and acceleration voltage of 0.2–30 kV. Regarding the working principle, when the sample surface is bombarded by the high energy electrons, >99% of the interaction between the incident electron energy will be converted into thermal energy, while the remaining 1% of the incoming electron energy will stimulate a secondary electron, the backscattering electron, and a characteristic X-ray.

## 3. Results and Discussion

### 3.1. Analysis of Compaction Test Law

As per the test procedures, 5-LZ-0 to 5-LZ-100 in Table 3 were successively subjected to compaction tests to obtain the maximum dry density and optimal water content of the mixture. The test results are shown in Table 5.

Table 5 shows that with the increase in the furnace slag substitution rate, the optimal water content of the mixture increased and the maximum dry density decreased. When the furnace slag substitution rate increased by 25%, the optimal water content of the mixture increased by ~1.3% on average; however, the maximum dry density decreased by ~2.6%. On the one hand, the surface of the furnace slag was rough, uneven, and porous, and it could absorb more water than coal gangue. The furnace slag, on the other hand, had a lower density than coal gangue. Therefore, when the 0–4.75 mm coal gangue was replaced by the 0–4.75 mm furnace slag, the optimal water content of the mixture increased and the maximum dry density decreased.

### 3.2. Analysis of the Unconfined Compressive Strength

The unconfined compressive strength was calculated using Equation (1):

(1) $$R_{c}=\frac{P}{A}\begin{array}{ccc} ; & & \end{array}A=\frac{\pi D^{2}}{4}$$

where *R*_c_ is the unconfined compressive strength (MPa) of the specimen, *P* is the maximum pressure of the specimen under failure (N), *A* is the cross-sectional area (mm^2^) of the specimen, and *D* is the diameter of the specimen (mm).

**Figure 3:**
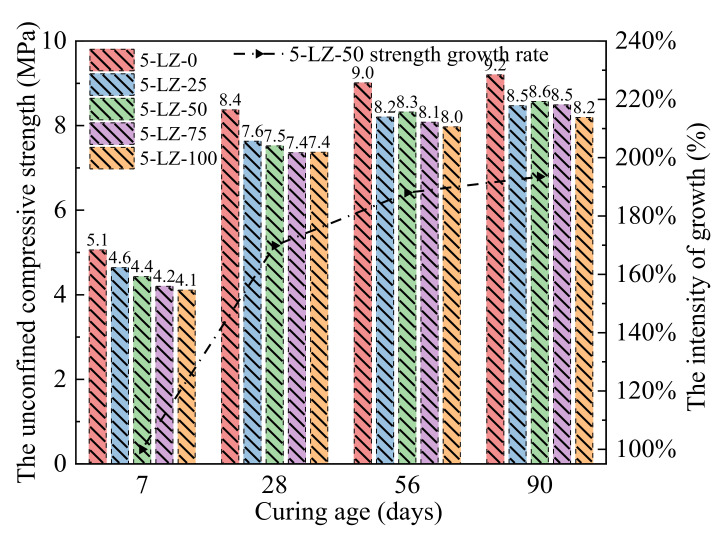
Results of the unconfined compressive strength tests at different furnace slag substitution rates.

The unconfined compressive strengths of mixtures listed in Table 3 at 7, 28, 56, and 90 days were measured and the results are shown in Figure 3.

As can be seen from Figure 3, the unconfined compressive strength of the specimen increased with treatment time. At different treatment periods, the unconfined compressive strength of 5-LZ-0 was higher than that of the specimen that was mixed with furnace slag, indicating that the furnace slag reduced the strength of the specimen. The furnace slag incorporation clearly deteriorated the early strength of the specimen. At a curing period of 7 days, the compressive strength of 5-LZ-0 was 8.2, 12.4, 16.8, and 18.5% higher than that of 5-LZ-25, 5-LZ-50, 5-LZ-75, and 5-LZ-100, respectively. The unconfined compressive strength of the specimen decreased as the substitution rate of the furnace slag increased because, on the one hand, the density of the furnace slag was less than that of the coal gangue. The density of the specimen gradually decreased as the substitution rate of the furnace slag increased. When the furnace slag substitution rate was set to 100%, the density of the specimen decreased by 10.6% compared to 5-LZ-0. When the cement mass fraction was constant, the actual mass of cement in each test piece was relatively reduced, and the early strength of the test piece was primarily derived from gel substances that were generated by the hydration reaction of cement, such as calcium silicate hydrate and calcium aluminate hydrate. After screening, however, the crushing value (the ability of a material to resist crushing) of furnace slag was lower than that of coal gangue, and the majority of the furnace slag particles had rough and irregular surface shapes, similar to the appearance of medium sand (i.e., sand with fine moduli of 3.0–2.3 and an average grain size of 0.5 mm). Compared with the 0–4.75 mm coal gangue, the cohesion and mechanical occlusions of the furnace slag and the cement–fly ash slurry were lower than those of the coal gangue. Therefore, with an increase in the furnace slag substitution rate, the compressive strength of the specimen gradually decreased. After a curing period of 28 days, the unconfined compressive strengths of the specimens significantly improved compared with those that were cured for seven days [30]. The growth rates of the compressive strengths of specimens with different mixing ratios were >60%, and the strength of 5-LZ-100 had the largest growth rate, reaching 79.5%. The primary reason for this was that with the extension of the treatment period, a large number of active substances, such as SiO_2_ and Al_2_O_3_, in the fly ash and furnace slag gradually underwent a secondary hydration reaction under the excitation of the cement hydration product, namely, Ca(OH)_2_, generating a large number of crystal substances, such as C-S-H and C-A-H. The chemical reaction formula is shown in Equation (2):

(2) $$\begin{array}{c} \mathrm{Ca}{\left(\mathrm{OH}\right)}_{2}+{\mathrm{SiO}}_{2}+H_{2}O\to \mathrm{CaO}\cdot {\mathrm{SiO}}_{2}\cdot 2H_{2}O\\ \mathrm{Ca}{\left(\mathrm{OH}\right)}_{2}+{\mathrm{Al}}_{2}O_{3}+H_{2}O\to \mathrm{CaO}\cdot {\mathrm{Al}}_{2}O_{3}\cdot 2H_{2}O \end{array}$$

The formation of these crystals made the interface between the furnace slag, coal gangue, and cement mortar closer and considerably improved the internal compactness and bonding strength, and, in turn, the overall strength of the specimen. At 56 and 90 days, the strength growth rate of the specimen significantly decreased, and its average value was 10.5. Moreover, with the increase in the furnace slag substitution rate, the strength growth of the specimen became more obvious. Because the amount of Ca(OH)_2_ and other active substances involved in the reaction gradually decreased as the degree of the number of secondary hydration reactions increased, the rate of the secondary hydration reaction decreased. Furthermore, the specimen’s water glue was relatively low, and the water required for the late hydration reaction was insufficient, resulting in the specimen’s strength growing slowly in the later stages. Because of the high concentrations of SiO_2_, Al_2_O_3_, and basic oxides in the furnace slag, the chemical activity of the furnace slag was higher than that of the coal gangue, and the slow secondary hydration reaction continued, even after an extended curing period [31,32]. With the increase in the furnace slag substitution rate, as active substances in the specimen increased, more water was required to reach the optimal water content, delaying the secondary hydration reaction to a later stage of the curing. Therefore, in the later stage of maintaining treatment, the adverse effect of the furnace slag addition on the specimen strength gradually decreased. At 90 days, the compressive strength of 5-LZ-0 was 7.9, 6.9, 7.8, and 11.0% higher than that of 5-LZ-25, 5-LZ-50, 5-LZ-75, and 5-LZ-100, respectively. In conclusion, the strength growth rate of the specimen significantly decreased as the curing period lengthened, and the strength growth rate of the specimen increased as the furnace slag replacement rate increased. Consequently, to ensure that the mixture’s 7-day unconfined compressive strength index met the appropriate specification, the utilization rate of furnace slag should be increased as much as possible. In practical engineering applications, the replacement rate of furnace slag should be between 50 and 75%.

**Figure 4:**
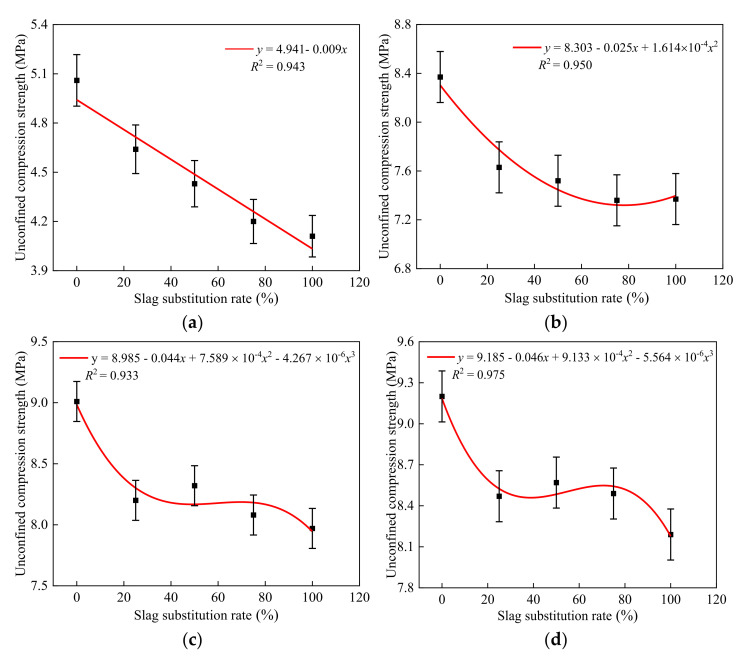
Fitting formulas of the unconfined compressive strength for different furnace slag substitution rates and curing periods: (**a**) 7 days, (**b**) 28 days, (**c**) 56 days, and (**d**) 90 days.

MATLAB was used to fit the unconfined compressive strengths of the specimens with different furnace slag substitution rates at curing periods of 7, 28, 56, and 90 days, as shown in Figure 4.

**Figure 5:**
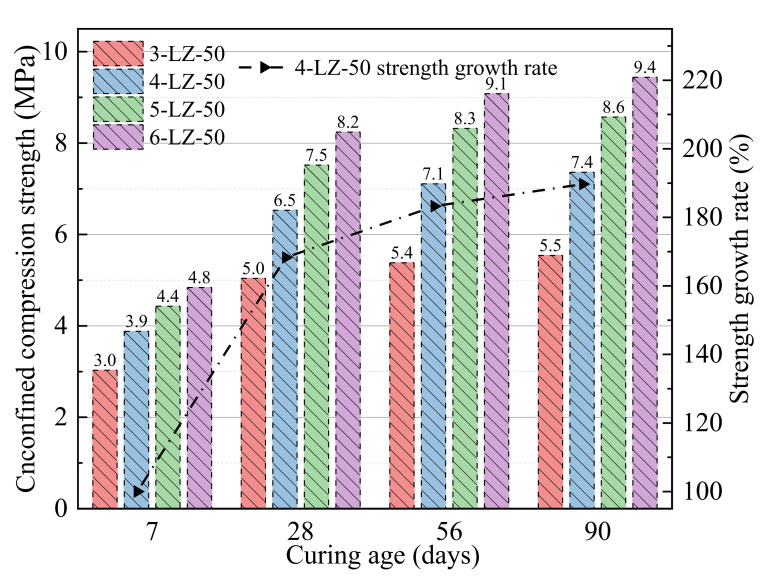
Unconfined compressive strength test results of specimens with different cement ratios.

As can be seen from Figure 4, the errors between the calculated values and experimental values of the fitting formulas were <5%, indicating a good degree of agreement. Thus, the fitting formulas can be used in engineering applications. The optimal furnace slag replacement ratio should be determined first before studying the strength development law of the specimen with different cement ratios. Three principles were followed in this study when determining the optimal furnace slag replacement rate. First, when the furnace slag was added, the specimen’s 7-day unconfined compressive strength loss rate was kept within 15%. Second, the specimen’s unconfined compressive strength decreased by <10% of its initial strength after 90 days. The third aim was to improve the furnace slag substitution rate as much as possible. As shown in Figure 3, when the furnace slag replacement rate was 25 or 50%, the specimen’s 7-day unconfined compressive strength loss rate was <15% [33]. At 90 days of curing, the unconfined compressive strength of 5-LZ-50 was slightly better than those with different furnace slag substitution rates, and the strength loss was <10%. Therefore, the optimal furnace slag replacement rate was selected as 50%, i.e., the value of x in Table 3 was 14 and the value of y was 50. As per the selected optimal furnace slag substitution rate of 50%, the unconfined compressive strengths of the specimens from 3-LZ-50 to 6-LZ-50 in Table 3 at 7, 28, 56, and 90 days were examined. The test results are shown in Figure 5.

**Figure 6:**
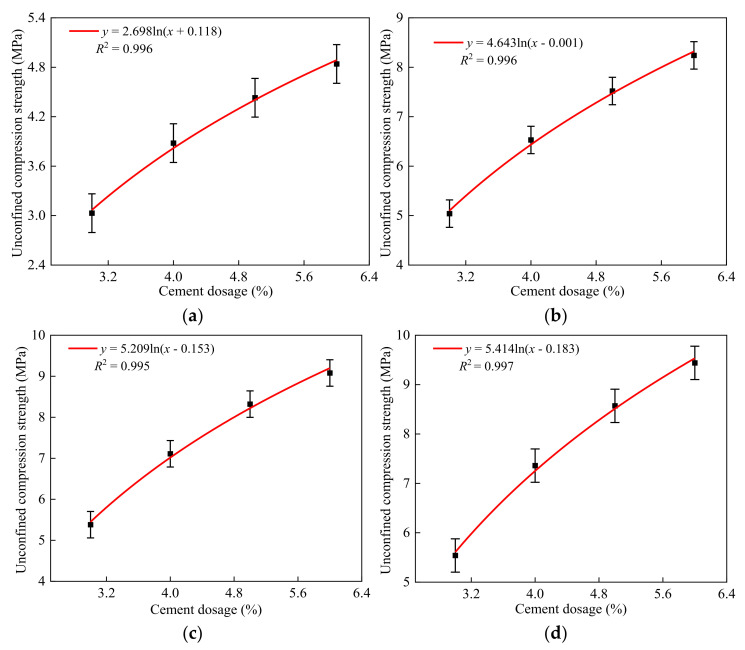
The fitting formulas of the unconfined compressive strengths for different cement content with different curing ages: (**a**) 7 days, (**b**) 28 days, (**c**) 56 days, and (**d**) 90 days.

Figure 5 shows that, with the increase in cement ratio, the strength growth of the specimens over different curing periods varied. When the cement ratio increased by 1% at 7 days, the unconfined compressive strength of the specimen increased by 0.9, 0.5, and 0.4 MPa. When the cement ratio increased from 3 to 4%, the unconfined compressive strength of the specimen obviously increased because, with the increase in cement ratio, the proportion of cement clinker minerals in the specimen increased. Furthermore, the content of Ca(OH)_2_ increased, which accelerated the hydration rate of the specimen in the early stage. When the cement ratio increased to 6%, the 7-day unconfined compressive strength growth of the specimen decreased to 0.4 MPa. This was because, as the cement ratio increased, the content of Ca(OH)_2_ in the specimen increased; however, the content of active substances such as SiO_2_ and Al_2_O_3_, which can participate in the secondary hydration reaction early in curing, decreased, and the secondary hydration reaction rate did not significantly increase, indicating that the specimen’s strength growth range slowed down. The specimen’s unconfined compressive strength significantly improved after 28 days compared to the age of 7 days. The average growth rate of the specimen’s compressive strengths at various cement ratios was 68.7%, which was primarily determined by the secondary hydration reaction and the microaggregate effect. The specimen’s strength growth rate noticeably decreased between 56 and 90 days, with an average value of 10.8%. The strength growth of the specimen became more visible as the cement ratio increased. This was because as the degree of secondary hydration reaction increased, the amount of Ca(OH)_2_ and other active substances that were involved in the reaction gradually decreased, lowering the rate of the reaction. However, with the increase in the cement ratio, the Ca(OH)_2_ content in specimens increased, and a relatively large amount of Ca(OH)_2_ in the specimens still had secondary hydration reactions with the fly ash, furnace slag, and coal gangue at a later curing period. Consequently, even after increasing the cement ratio, the specimens maintained a relatively high strength growth rate in the later stages of curing. The results of the tests demonstrated that the appropriate dosage of cement had a clear effect on the specimen’s early strength. When the cement dosage was >5%, the effect of increasing the cement dosage on the early strength improvement of the specimen was not obvious; however, it showed a relatively obvious strength growth at the later stage of curing. Consequently, when the early strength requirements of the cement and fly ash materials are high, measures such as improving raw material technical indexes and optimizing grading design should be implemented. Only increasing the cement ratio could improve the early unconfined compressive strength of the specimens. MATLAB was used to fit the unconfined compressive strength of the specimens with different cement ratios at 7, 28, 56, and 90 days, as shown in Figure 6.

As can be seen from Figure 6, the errors between the calculated values and experimental values of the fitting formulas were <5%, indicating a good degree of agreement. The strength fitting formula at each age can be a good relationship between the replacement rate of response furnace slag and the compressive strength. This formula can be used as a theoretical basis for practical engineering applications.

### 3.3. Analysis of the Splitting Test Law

Equation (3) shows the calculation method for the splitting strength of the specimen with a diameter of 150 mm:

(3) $$R_{i}=0.004178\frac{p}{h}$$

where *R*_i_ is the splitting strength (MPa) of the specimen, *p* is the maximum pressure of specimen failure (N), and *h* is the height (mm) of the specimen after immersion.

**Figure 7:**
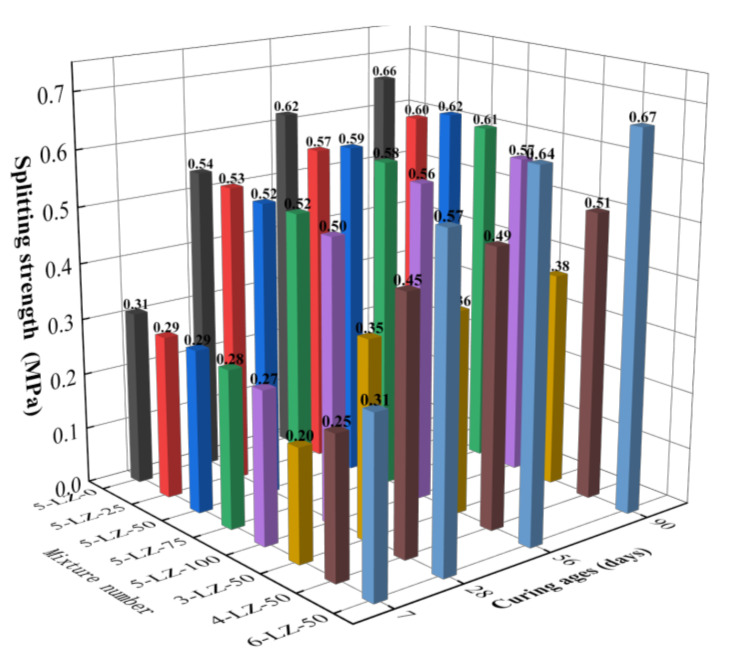
Results of the splitting test for different ages.

The splitting test was performed for the eight mixing ratios listed in Table 3, and the test results are shown in Figure 7.

As can be seen from Figure 7, with the increase in cement ratio, the splitting strength of the specimen showed an increasing trend. The fracture strength of the specimen increased with treatment age and followed the same growth pattern as the unconfined compressive strength. The splitting strength of 5-LZ-0 was greater than that of the specimens that were mixed with cement and furnace slag, indicating that the incorporation of furnace slag reduced the splitting strength of the specimens.

At the age of 7 days, with the increase in the cement ratio and decrease in the furnace slag substitution rate, the splitting strength of the specimen demonstrated an increasing trend. The primary causes were the same as in the unconfined compressive strength test. When compared to 7 days, the splitting strength of the specimen at 28 days had significantly improved, where the average growth rate of the splitting strength of the specimen was 81.0%. On the one hand, the average particle size of the fly ash particles was considerably smaller than that of the coal gangue and furnace slag, allowing for a more evenly filled accumulation system among the aggregates and a more sufficient secondary hydration reaction. On the other hand, with the extension of the curing age, active substances in the fly ash and furnace slag gradually underwent secondary hydration reactions, which consumed Ca(OH)_2_ in the specimen, restrained the growth of Ca(OH)_2_ grains, and reduced the thickness of the interface transition zone. With the continuous consumption of Ca(OH)_2_, the amount of gel material generated increases, further improving the splitting strength of the specimen. At 56 and 90 days, the splitting strengths of the specimen with a furnace slag replacement rate of 50% were slightly better than those of the specimens with furnace slag replacement rates of 25, 75, and 100%. The strength growth law from 5-LZ-25 to 5-LZ-100 showed that, with the increase in furnace slag substitution rate, the splitting strength of the specimen first increased and then decreased, and the change in this trend was determined by the failure mode of the splitting test specimen. The fracture and compressive strength variations and the strength formation mechanism of the specimens were similar; however, their failure modes were different. When the furnace slag substitution rate increased from 25 to 50%, the interface transition zone of the microscopic pore structure increased, the continuous deflection of microcracks consumed more fracture energy, and the splitting tensile strength improved. However, as the substitution rate of the furnace slag increased, the amount of 0–4.75 mm coal gangue decreased, and the pores of furnace slag could not be filled with cement, fly ash, and coal gangue fine particles, resulting in specimen compaction. Furthermore, as the substitution rate of the furnace slag increased, the density of the specimen decreased and the proportion of cement clinker in the specimen decreased, reducing the number of secondary hydration reactions. Furthermore, the proportion of unreacted furnace slag and fly ash increased, reducing the splitting strength of the specimen.

Thus, the splitting strength of coal gangue that was stabilized by cement, fly ash, and furnace slag was a comprehensive reflection of the coal gangue strength, hydration reaction degree of the cementing material, and the internal cohesion of the mixture. Because the strength of the furnace slag aggregate was lower than that of the coal gangue, the splitting strengths of the specimens mixed with furnace slag were generally lower than those of 5-LZ-0. Furthermore, the porosity of furnace slag promoted the infiltration of the cement–fly ash slurry into the aggregate and improved the specimen’s compactness and stability. Moreover, the active substances in the furnace slag and fly ash underwent a secondary hydration reaction in the middle and late stages of curing, thus producing many C-A-H and C-S-H cementing products that improved the internal structure of the interface area of each aggregate and increased the specimen’s strength. When the cement ratio was 4% and the furnace slag replacement rate was 50%, the 90-day splitting strength of the specimen was >0.4 MPa, which met the technical indexes of highway pavement base materials in China.

### 3.4. Analysis of Unconfined Compressive Strength and Splitting Strength

From the abovementioned test results, the unconfined compressive strength of the test specimen was positively correlated with the splitting strength, which is important since the relationship between them is a hot topic for researchers. However, few studies exist on the relationship between the unconfined compressive strength of the pavement base material and its splitting strength. Therefore, using Equation (4), the relationship between the unconfined compressive strength and the splitting strength of the pavement base material was obtained:

(4) $$f_{t}=k{\left(f_{c}\right)}^{n}$$

where *f*_t_ is the concrete splitting tensile strength (MPa), *f*_c_ is the compressive strength of the concrete cylinder (MPa), and the values of *k* and *n* were obtained using nonlinear regression analysis.

**Figure 8:**
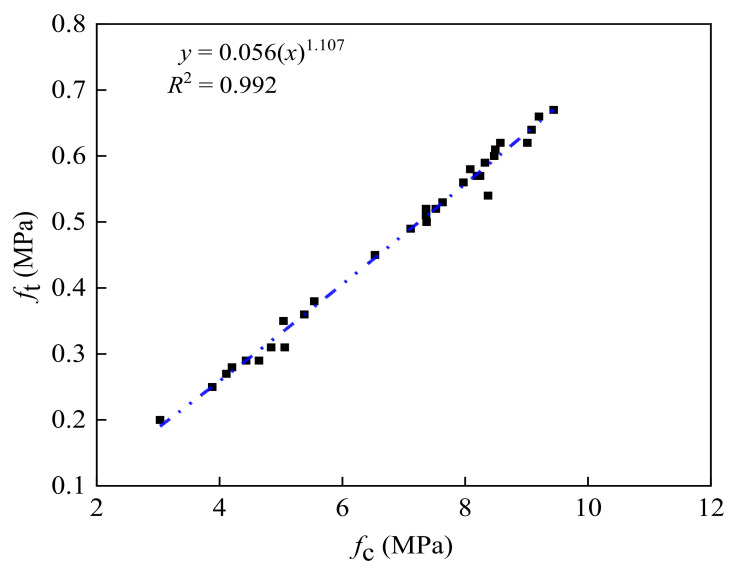
Relationship between the unconfined compressive strength and the splitting strength.

The regression was analyzed in the form of the power function. Figure 8 shows the relationship between the unconfined compressive strength and the splitting strength.

The regression equation showing the relationship between the unconfined compressive strength and splitting strength was given by

(5) $$\begin{array}{c} f_{t}=0.056{\left(f_{c}\right)}^{1.107}\\ R^{2}=0.992 \end{array}$$

As can be seen from Figure 8, Equation (5) shows good agreement with these test results; however, the power function had a better correlation with the test results. Therefore, to guide practical engineering applications, it is recommended to use the power function to predict the splitting strength of specimens for different curing periods.

### 3.5. Analysis of Triaxial Test Law

#### 3.5.1. Failure Analysis

**Figure 9:**
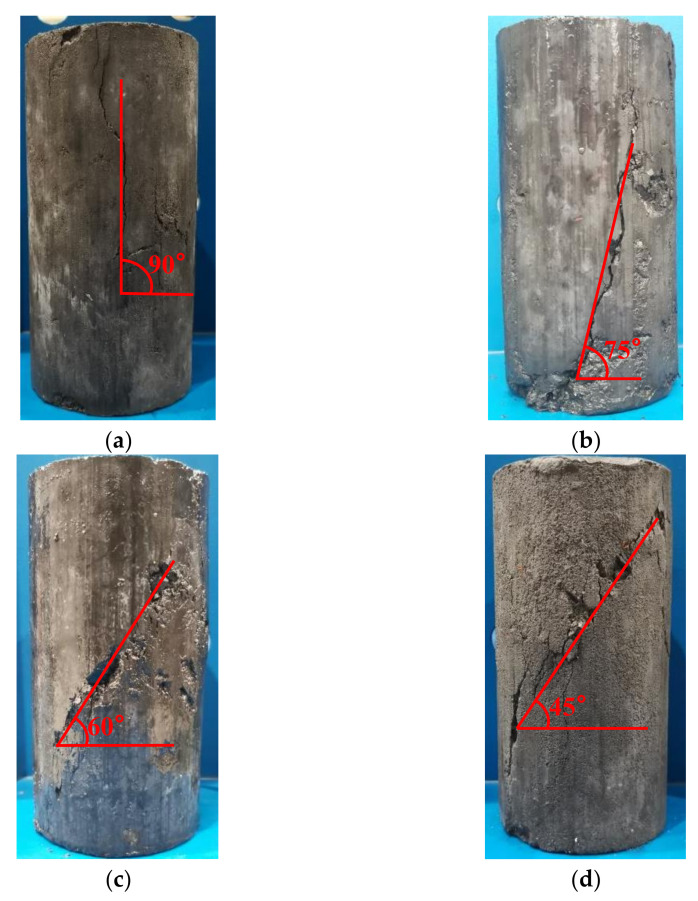
Failure modes of a specimen under different confining pressures: (**a**) *σ*_2_ = *σ*_3_ = 0, (**b**) *σ*_2_ = *σ*_3_ = 0.5 MPa, (**c**) *σ*_2_ = *σ*_3_ = 1 MPa, and (**d**) *σ*_2_ = *σ*_3_ = 1.5 MPa.

Figure 9 shows the typical failure patterns of a specimen under three-direction stress. Observing the failure mode of the specimen revealed that it was unaffected by the substitution rate of furnace slag and the cement ratio and was primarily related to the confining pressure. Vertical cracks appeared in the middle of the specimens, almost in the loading direction, when the confining pressure value was 0. With the increase in load, the cracks expanded and developed into one or more penetrating cracks, causing the specimens to fail.

The following are shown in Figure 9: (1) When the confining pressure was 0.5 MPa, the crack direction of the specimen was no longer in the direction of the principal stress but was at an angle of ~15° with the principal crack. Shear failure dominated the failure surface. (2) When the confining pressure value was 1 MPa, the angle between the direction of the main crack and the direction of the main stress was ~30°, the main crack ran through each of the entire specimens, and the concrete on both sides of the crack was relatively staggered because of shear stress action. (3) When the confining pressure was 1.5 MPa, the included angle of the specimens between the main crack direction and the main stress direction expanded to ~45°, and part of the gangue aggregate on the shear surface was cut off, accompanied by falling specimen pieces.

#### 3.5.2. Stress–Strain Curve Analysis

**Figure 10:**
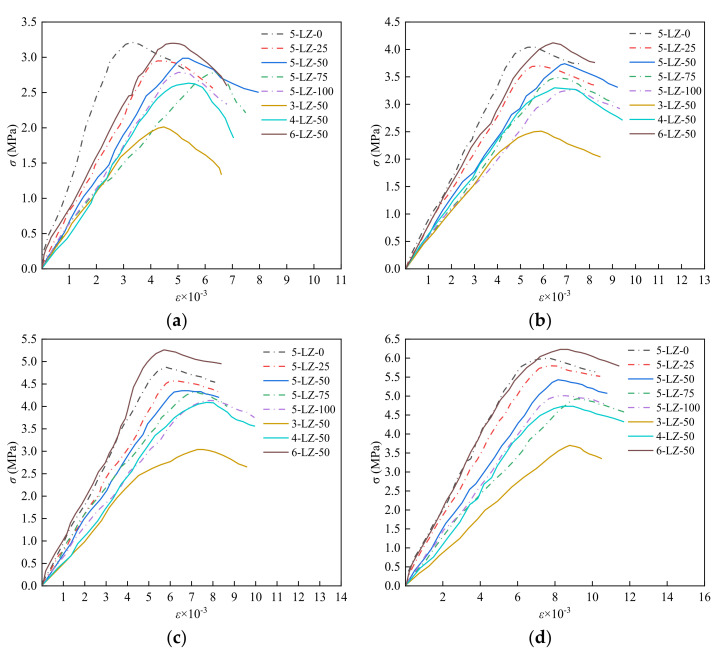
Stress–strain curves of the specimens: (**a**) *σ*_2_ = *σ*_3_ = 0, (**b**) *σ*_2_ = *σ*_3_ = 0.5 MPa, (**c**) *σ*_2_ = *σ*_3_ = 1 MPa, and (**d**) *σ*_2_ = *σ*_3_ = 1.5 MPa.

After the load–displacement curve was collected by the tester and calculated using Equations (4) and (5), it was converted into stress and strain values, and the *σ*–*ε* curve was obtained, as shown in Figure 10:

(6) $$\sigma =\frac{F}{A_{1}}\begin{array}{cc} & \varepsilon =\frac{\Delta l}{h} \end{array}\begin{array}{cc} & A_{1} \end{array}=\frac{A}{1-\varepsilon }$$

where *σ* is the axial stress (MPa), *F* is the axial load (N), *A*_1_ is the effective cross-sectional area of the specimen (mm^2^), *ε* is the axial strain, Δ*l* is the axial displacement (mm), *h* is the initial height of the specimen (mm), and *A* is the initial cross-sectional area of the specimen (mm^2^).

Figure 10 shows that the σ–ε curves of the specimens under the triaxial stress state significantly changed with the increase in confining pressure. The *σ*–*ε* curves of the specimens could be roughly divided into three stages: (1) The elastic stage—At the beginning of loading, with the increase in load, the σ–ε curves of the specimens were almost linear, showing obvious elastomer properties. (2) Elastic–plastic stage—With the increase in load, the strain increase rates of the specimens were significantly higher than that of the elastic stage, and the slopes of the σ–ε curves of the specimens gradually decreased. (3) The stage of destruction—The curves of the specimens began to decrease after reaching the peak stress as the load was increased further; however, there was no obvious peak, demonstrating the ductility of the semi-rigid base failure. The peak stress of the specimens was lower with furnace slag than without, but the corresponding peak strain was higher with furnace slag. The incorporation of furnace slag reduced the strength of the specimens while increasing their ductility to a certain extent. When the confining pressure was 0, the specimens’ peak stress was less than when the confining pressure was present, and the slope of the failure stage curve was steeper. When the confining pressure was 0.5 MPa, both the peak stress and peak strain of the specimens tended to increase; furthermore, the descending slope of the failure stage curve slightly decreased. When the confining pressure was 1 MPa, the peak stress and peak strain of the specimens increased further, and the descending slope of the curve in the failure stage became more gentle. When the confining pressure was 1.5 MPa, the downward trend of the specimens in the failure stage was gentler than that when the confining pressure was 0, showing better ductility.

#### 3.5.3. Stress Peak Law Analysis

**Figure 11:**
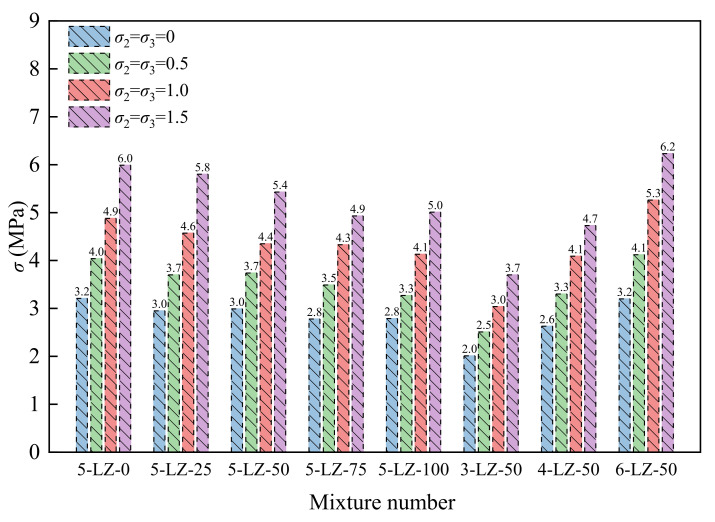
Peak stress of specimens under different confining pressures.

As per the full *σ*–*ε* curve of the specimens in Figure 10, the peak stress of the specimens under different confining pressures could be obtained, as shown in Figure 11.

**Figure 12:**
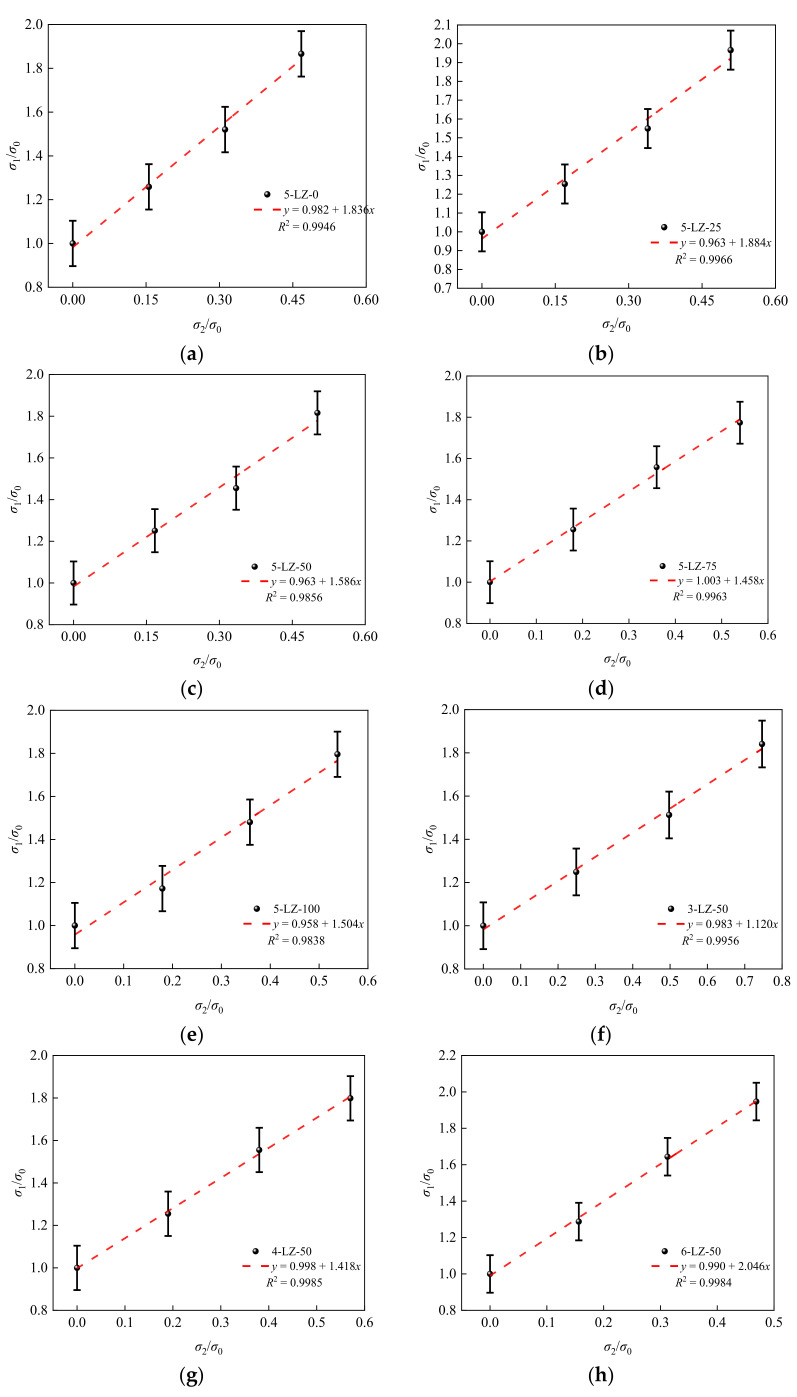
Relationship between the peak stress and confining pressure: (**a**) 5-LZ-0, (**b**) 5-LZ-25, (**c**) 5-LZ-50, (**d**) 5-LZ-75l, (**e**) 5-LZ-100, (**f**) 3-LZ-50, (**g**) 4-LZ-50, and (**h**) 6-LZ-50.

Figure 11 shows that, with the increase in confining pressure, the peak stress of all specimens demonstrated an increasing trend and was significantly higher than that under the uniaxial stress state. This was because the confining pressure reduced the transverse deformation of the specimen while restricting the expansion of the internal microcracks, resulting in a significant increase in the specimen’s peak stress. As per the general trend, as the substitution rate of furnace slag increased, the peak stress of the specimens decreased. The peak stress of the specimens increased with the increase in cement ratio. Its causes and rules of change were similar to those of the unconfined compressive strength. As per the reference, the dimensionless treatment was performed on the test results, and the relationship between the peak stress and confining pressure of the specimen was obtained through analysis, as shown in Figure 12.

By fitting the relationship between the peak stress and confining pressure of the specimen, the unified strength calculation formula of the specimen was obtained [34]:

(7) $$\frac{\sigma_{1}}{\sigma_{0}}=1+A\frac{\sigma_{2}}{\sigma_{0}}$$

where *σ*_1_ is the peak stress of the specimen under three-direction stress (MPa), *σ*_0_ is the peak stress of the specimen under uniaxial stress (MPa), *σ*_2_ is the confining pressure (MPa), and A is a coefficient that is related to the furnace slag substitution rate *α* and cement ratio *β*.

**Figure 13:**
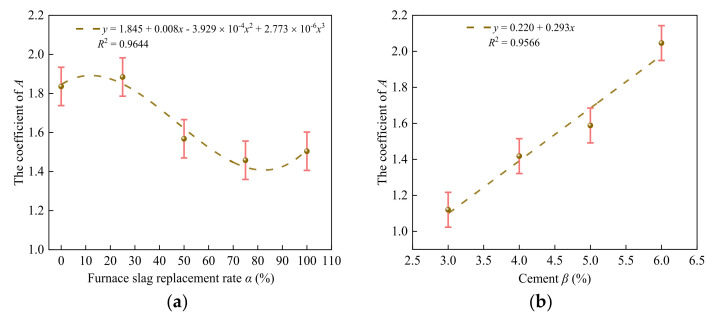
Fitting results of the furnace slag substitution rate *α*, cement ratio *β*, and coefficient *A*: (**a**) relation between the substitution rate *α* and coefficient *A* of the furnace slag and (**b**) relationship between the cement ratio *β* and coefficient *A*.

Figure 13 shows the relationships between coefficient A and the furnace slag substitution rate α and cement ratio *β*.

From Figure 13a, Equation (8) was obtained:

(8) $$A=1.845+0.008x-3.929\times 10^{-4}x^{2}+2.733\times 10^{-6}x^{3}$$

Substituting Equation (8) into Equation (7) to obtain the relationship between the peak stress and confining pressure of specimens at different furnace slag substitution rates gave

(9) $$\frac{\sigma_{1}}{\sigma_{0}}=1+(1.845+0.008x-3.929\times 10^{-4}x^{2}+2.733\times 10^{-6}x^{3})\frac{\sigma_{2}}{\sigma_{0}}$$

From Figure 13b, Equation (11) was obtained:

(10) A=0.220+0.293x

By substituting Equation (10) into Equation (7), we obtained the relationship between the peak stress and confining pressure of specimens with different cement ratios:

(11) $$\frac{\sigma_{1}}{\sigma_{0}}=1+(0.220+0.293x)\frac{\sigma_{2}}{\sigma_{0}}$$

Figure 13 shows that the fitting curve was in good agreement with the test results; therefore, Equations (10) and (12) can reflect the relationship between the furnace slag substitution rate, cement ratio, peak stress, and confining pressure.

### 3.6. Strength Formation Mechanism

**Figure 14:**
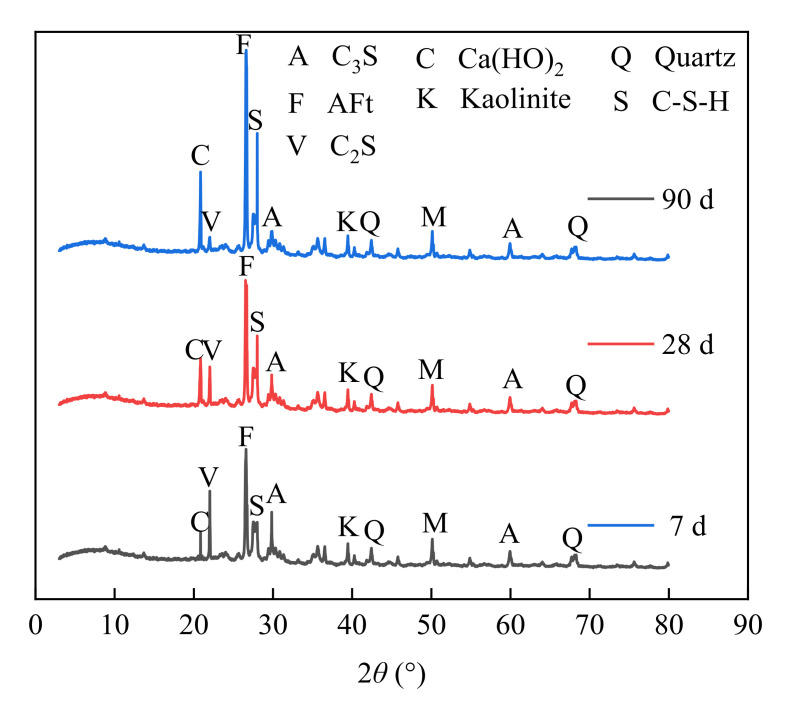
XRD detection at different ages.

**Figure 15:**
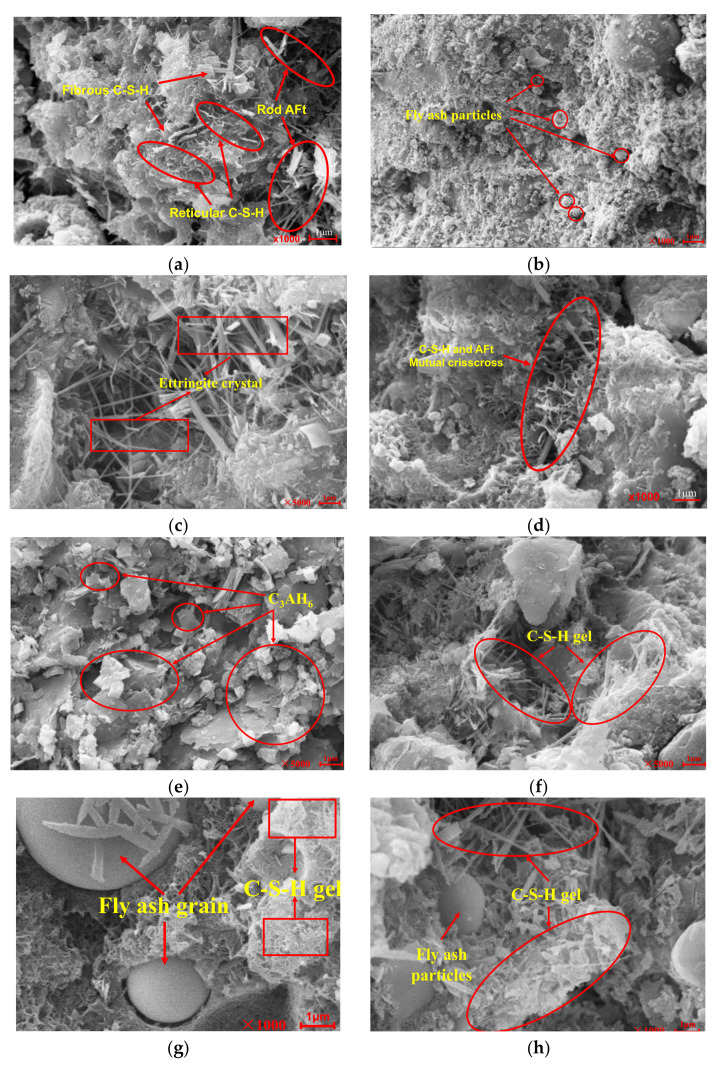

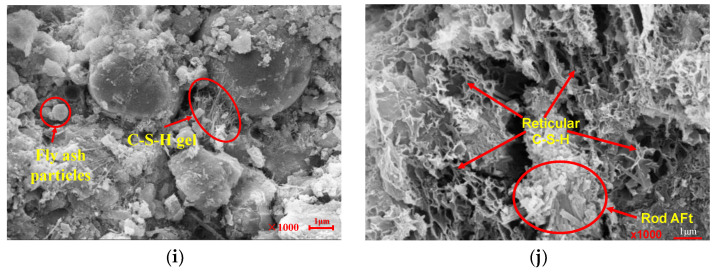
Microstructure of the mixture: (**a**–**c**) curing time of 7 days, (**d**–**f**) curing time of 28 days, and (**g**–**j**) curing time of 90 days.

The strength formation mechanism of the cement–fly-ash-stabilized furnace slag–coal gangue mixture was similar to that of concrete mixed with fly ash. Under the action of water, complex chemical reactions occur within the mixture to generate various substances, such as C-S-H, C-A-H, and ettringite (AFt), which improves the compactness and strength of the mixture. We selected 5-LZ-50 as a representative sample and dried it. Then, the samples at 7, 28, and 90 days of age were tested using XRD and scanning electron microscopy (SEM), where the changes of hydration products at different ages were analyzed and their micromorphologies were observed, as shown in Figure 14 and Figure 15, respectively.

From Figure 14, the 5-LZ-50 sample showed different XRD composition results at different ages of 7, 28, and 90 days. It can be seen from the figure that at 7 days, the contents of AFt, C-S-H, and Ca(OH)_2_ in the mixture were low, but the contents of C_2_S and C_3_S were high. With the increase in age, the amount of Ca(OH)_2_ in the mixture increased. Due to the secondary hydration of fly ash, the contents of AFt, C-S-H, and Ca(OH)_2_ in the mixture continued to increase, the strength of the mixture was further increased in the later stage due to the mutual interleaving, and part of C_2_S and C_3_S in the mixture was consumed under the secondary hydration in the later stage. Therefore, it can be seen from Figure 14 that the contents of AFt, C-S-H, and Ca(OH)_2_ in the later stages of the mixture were increasing, and the contents of C_2_S and C_3_S were decreasing.

From the microscopic point of view, the strength formation mechanism of the coal gangue mixture that was stabilized by cement and fly ash could be explained as the physical filling of each admixture and the development of hydration products from an amorphous gel to highly crystalline material. As shown in Figure 15, the colloidal cement and fly ash mortar objects in the mixture primarily comprised amorphous coliform C-S-H, needle-stick structure ettringite (AFt), unhydrated cement clinker particles, furnace slag particles, and fly ash particles. The primary components were C-S-H, AFt, and fly ash particles, which account for ~80% of the total volume [35,36]. The C-S-H was amorphous, granular, and fibrous, with networks and dense sheets forming. Generally, the appearance of C-S-H is related to its growth space and environment. In addition to the various forms mentioned above, C-S-H can take the form of a sheet, needle, rod, or petal, as shown in Figure 15f. Ettringite crystals, in general, are hexagonal prismatic crystals whose morphology is related to the growth space and ion supply, as shown in Figure 15c. However, with an increase in the treatment period, the reticulated C-S-H gel material and the needle-like AFt crystal intersected and grew and gradually connected as a whole, as shown in Figure 15i,j.

Figure 15 shows that, with an increase in the treatment age, the amount and form of hydration products significantly changed. After the treatment for seven days, as shown in Figure 15a–c, the products of the specimens were primarily needle-like particles and a reticulated flocculent gel. It had more pores and lower compactness when cured for 28 days, as shown in Figure 15d–f. When the treatment lasted 90 days, the specimen’s compaction increased even more, and the needle- and rod-like gels developed through each other, thus forming a C-S-H gel network. These gels tightly wrapped the fly ash particles, thus forming a relatively dense overall structure, as shown in Figure 15g–j. The reason for this was that as the treatment time was extended, the active substances of fly ash and furnace slag gradually reacted with the Ca(OH)_2_ generated by cement hydration to generate many C-S-H and C-A-H gels, which gradually grew and developed into the micropore space inside the specimen and gradually increased the specimen’s compactness and internal cohesion [37], indirectly improving the mixture’s strength. Generally, the hydration reaction inside the specimen leads to higher compactness and strength [38]. Therefore, the hydration degree of the test piece is guaranteed under appropriate curing conditions.

According to the test and microscopic detection results, the strength formation mechanism of the specimen mainly included the following three aspects. The first one was that when the furnace slag, cement, fly ash, and coal gangue were fully mixed, the phase composition in the pore of furnace slag involved the solid phase (cement, fly ash, and fine particle furnace slag and coal gangue), liquid phase (water molecules), and the gas phase (the unfilled area in the furnace slag pores). With the increase in the treatment time, cement and fly ash in the furnace slag hole gradually formed the C-S-H gel, which filled the furnace slag pores and improved the bearing capacity of the furnace slag. Second, cement, fly ash, and coal gangue with fine particles could fill in between the furnace slag particles and coal gangue, forming a microfilling effect and improving the compactness and stability of the cement–slurry interface. The third aspect was the low strength of the slag aggregate itself, but under the comprehensive filling effect of cement, fly ash, and coal gangue powder and its own certain activity, the slag substitution rate had a positive impact on the compressive strength of the test part in the later period of the curing.

## 4. Conclusions

(1) With the increase in the furnace slag substitution rate, the optimal water content of the specimen increased and the maximum dry density decreased. The addition of the furnace slag reduced the unconfined compressive strength of the specimen, where the greater the substitution rate of the furnace slag, the lower the unconfined compressive strength of the specimen. However, with an extended curing period, the effect of the furnace slag on the unconfined compressive strength of the specimen gradually weakened. With the increase in cement ratio, the unconfined compressive strength of the specimen increased. Considering the principle of mechanical properties and economic concerns, it is suggested that the replacement rate of furnace slag in actual construction should be about 50% and should not exceed 75%.
(2) The results showed that the power function agreed well with the experimental results and can be used in practical engineering applications.
(3) The triaxial test results were analyzed in terms of the failure mode, stress–strain relationship curve, peak stress, and failure criterion of the specimens. The results showed that (i) under the triaxial stress, the failure mode of the specimens was greatly affected by confining pressure and they showed typical shear failure, and (ii) according to the test results, the formulas for the relationship between the furnace slag substitution rate, cement ratio, peak stress, and confining pressure were obtained.
(4) The SEM results showed that the hydration products of the mixture included colloidal C-S-H, needle-like ettringite (AFt), unhydrated cement clinker particles, and fly ash particles. The strength of the mixture was a comprehensive embodiment of the microaggregate effect, secondary hydration reaction, and the characteristics of the materials.

## Figures and Tables

**Table 1:** Chemical composition (mass fraction (%)) of furnace slag and fly ash.

Raw Materials	SiO_2_	Al_2_O_3_	Fe_2_O_3_	CaO	MgO	K_2_O	Na_2_O	TiO_2_	SO_3_	Loss on Ignition
Furnace slag	47.47	22.69	8.77	8.57	1.84	2.23	1.09	1.56	1.21	4.52
Fly ash	45.16	32.91	7.47	4.95	1.20	2.12	0.78	1.77	0.80	2.63
Coal gangue	54.65	28.28	4.81	5.06	1.03	3.02	0.32	1.52	0.64	1.51

**Table 2:** Basic properties of the furnace slag and coal gangue.

Sample	Particle Size (mm)	Crush Value (%)	Apparent Density (g·cm^−3^)	Packing Density (g·cm^−3^)	Water Absorption (%)
Furnace slag aggregate	0–2.36	/	2.353	0.786	11.1
	2.36–4.75	38.4	2.414	0.758	
Grade coal gangue	19.0–37.5	/	2.568	1.396	3.0
	9.5–19	29.5	2.574	1.461	
	4.75–9.5	/	2.562	1.432	
	0–4.75	/	2.543	1.468	

**Table 3:** Screening results of natural slag.

Screening Size (mm)	Screening Quality (g)	Grade Screening (%)	Accumulated Screening (%)	By Percentage (%)
>19	14.1	4.7	4.7	95.3
16	16.8	1.9	6.6	93.4
13.2	32.5	3.7	10.3	89.7
9.5	38.9	4.4	14.7	85.3
4.75	82.2	9.3	24.0	76.0
2.36	67.9	7.6	31.6	68.4
1.18	44.6	5.0	36.6	63.4
0.6	59.9	6.7	43.3	56.7
0.075	99.4	11.2	97.4	2.6
<0.075	23.2	2.6	100	0

**Table 4:** Mixing ratio.

Mixture Number	Mass Fraction of the Material (%)						
	Coal Gangue Grading (mm)				Furnace Slag (mm)	Fly Ash	Cement
	16–26.5	9.5–16	4.75–9.5	0–4.75	0–4.75		
5-LZ-0	18	19	15	28	0	15	5
5-LZ-25	18	19	15	21	7		5
5-LZ-50	18	19	15	14	14		5
5-LZ-75	18	19	15	7	21		5
5-LZ-100	18	19	15	0	28		5
3-LZ-y	18	19	16	28-x	x		3
4-LZ-y	18	19	17	28-x	x		4
6-LZ-y	18	19	18	28-x	x		6

Note: Take 5-LZ-50 as an example to illustrate the numbering method of the mixture. The 5 means the mass fraction of cement was 5% and LZ-50 means the mass fraction of the 0–4.75 mm furnace slag replacing the 0–4.75 mm coal gangue was 50%. The value of x was determined by the unconfined compressive strength test from 5-LZ-0 to 5-LZ-100, and *y* = 100*x*/28.

**Table 5:** Specimen optimal moisture content and maximum dry density.

Mix Number	Optimal Moisture Content (%)	Maximum Dry Density (g·cm^−3^)
5-LZ-0	8.7	2.018
5-LZ-25	9.7	1.966
5-LZ-50	10.9	1.910
5-LZ-75	12.2	1.853
5-LZ-100	13.6	1.804

## Data Availability

All of the data are available.

## References

[1] Hubiao Z., Fei T.Y., Changyu Y., Shudong H., Hongbo L. (2021). Study on road performance of fly ash and gangue mixture. Ningxia Eng. Technol..

[2] Sun H., Xu Q., Yan P., Yin J., Lou P. (2020). A Study on Axial Compression Performance of Concrete-Filled Steel-Tubular Shear Wall with a Multi-Cavity T-Shaped Cross-Section. Energies.

[3] Ding F.X., Wang W.J., Lu D.R., Liu X.M. (2020). Study on the behavior of concrete-filled square double-skin steel tubular stub columns under axial loading. Structures.

[4] Lu D.R., Wang W.J., Ding F.X., Liu X.M., Fang C.J. (2021). The impact of stirrups on the composite action of concrete-filled steel tubular stub columns under axial loading. Structures.

[5] Wang X., Yang X., Ren J., Han N., Xing F. (2020). A novel treatment method for recycled aggregate and the mechanical prop-erties of recycled aggregate concrete. J. Mater. Res. Technol..

[6] Reis G.S.D., Quattrone M., Ambrós W.M., Grigore Cazacliu B., Hoffmann Sampaio C. (2021). Current Applications of Recycled Aggregates from Construction and Demolition: A Review. Materials.

[7] Revilla-Cuesta V., Ortega-López V., Skaf M., Manso J.M. (2020). Effect of fine recycled concrete aggregate on the mechanical behavior of self-compacting concrete. Constr. Build. Mater..

[8] Agrela F., Díaz-López J.L., Rosales J., Cuenca-Moyano G.M., Cano H., Cabrera M. (2021). Environmental assessment, mechanical behavior and new leaching impact proposal of mixed recycled aggregates to be used in road construction. J. Clean. Prod..

[9] Zhu K., Ma X., Yao L., Zhao L., Luo C. (2021). Effect of Polypropylene Fiber on the Strength and Dry Cracking of Mortar with Coal Gangue Aggregate. Adv. Mater. Sci. Eng..

[10] Guangyu Y., Mingkai Z., Xiao C. (2021). Application of gangue aggregate pavement. J. Wuhan Univ. Technol. (Transp. Sci. Eng. Ed.).

[11] Zhang Y., Meng W., Zhang Z. (2013). Experimental study of indirect tensile strength of calcareous coal gangue mix-ture. World J. Eng..

[12] Liao J.G., Ma Q., Zhang Y.S., Song Z.Y., Liu K.H., Hu Y.W. (2012). Research on Solid Wastes of Gangue, Slag, Fly Ash Used as Cement Composite Mixing Materials. Adv. Mater. Res..

[13] Zhou M., Li Z.G., Wu Y.Q., Zhang X.F., Ai L. (2010). Study on lime-fly ash-cement stabilized gangue mixture. J. Build. Mater..

[14] Chalangaran N., Farzampour A., Paslar N., Fatemi H. (2021). Experimental investigation of sound transmission loss in concrete containing recycled rubber crumbs. Adv. Concr. Constr..

[15] Jisheng Q., Yunxian Z., Minhuang W. (2020). Damage characteristics and constitutive relationship of coal gangue con-crete under freeze-melting cycle. Int. J. Civ. Environ. Eng..

[16] Chalangaran N., Farzampour A., Paslar N. (2020). Nano Silica and Metakaolin Effects on the Behavior of Concrete Containing Rubber Crumbs. CivilEng.

[17] Wensheng S. (2011). Performance Control for Highway Coal Gangue Filling Subgrade Road.

[18] Yan Z., Hassan B., Rong Z. (2019). Evaluation for the Leaching of Cr from Coal Gangue Using Expansive Soils. Processes.

[19] Fan X.H., Xue Z.H. (2020). Study on road performance of recycled aggregate from construction waste. Highway.

[20] Qiu J.S., Zhou Y.S., Wang M.H., Houbo W. (2020). Damage characteristics and constitutive relationship of gangue concrete under freeze-thaw cycles. J. Civ. Environ. Eng..

[21] Ozcan T., Gokhan G., Zaimoglu A.S. (2014). Effect of Bentonite, Fly Ash and Silica Fume cement injections on uniaxial compressive strength of granular bases. KSCE J. Civ. Eng..

[22] Khatib J.M., Mangat P.S., Wright L. (2014). Pore size distribution of cement pastes containing fly ashgypsum blends cured for 7 days. KSCE J. Civ. Eng..

[23] Xiangdong Z., Kun K. (2017). Study on base strength and thermal conductivity of cement stable cinder surface. Non-Met. Mine.

[24] Changxuan H. (2020). Fatigue characteristics of cement-stabilized slag gravel mixture. Highw. Traffic Technol..

[25] Ministry of Housing and Urban-Rural Development of the People’s Republic of China.

[26] Meng S., Liu J., Zhong P., Wang G., Wang C. (2021). Study on granulation process of uranium containing Coal-fired Fly Ash. Uranium Min. Metall..

[27] Peng G. (2020). Research and Application of Cement Stable Gasized Porous Slag Pavement.

[28] Ministry of Communications of the People’s Republic of China (2009). Test Regulations for Inorganic Bonded Stability Materials of JTG E51–2009, Highway Engineering.

[29] Jia L., von Deluan A. (2021). A multiscale coupled finite element analysis method considering the microscopic motion of soil particles. Rock Earth Mech..

[30] Shabab M.E., Shahzada K., Gencturk B., Ashraf M., Fahad M. (2016). Synergistic effect of fly ash and bentonite as partial replacement of cement in mass concrete. KSCE J. Civ. Eng..

[31] Kheradmand M., Abdollahnejad Z., Pacheco-Torgal F. (2018). Shrinkage Performance of Fly Ash Alkali-activated Cement Based Binder Mortars. KSCE J. Civ. Eng..

[32] Nordine L., Van-Huong N., Pierre M. (2017). The effect of the partial cement substitution with fly ash on Delayed Ettringite Formation in heat-cured mortars. KSCE J. Civ. Eng..

[33] Farzampour A. (2019). Compressive Behavior of Concrete under Environmental Effects.

[34] Avic D., Zhen H. (2021). Numerical Implementation and Application of a Corcorner Model for generalized double-shear stress criterion. J. Rock Mech. Eng..

[35] Ding S., Niv D.T., Wang J.B. (2015). Experimental Study on Micro-structure and Mechanical Properties of Shotcrete with Fly Ash. Bull. Chin. Ceram. Soc..

[36] Li H., Wang J.B., Guo Q.J. (2020). Mechanical Properties of Recycled Aggregate Concrete with Mineral Admixture. Bull. Chin. Ceram. Soc..

[37] Ding F.X., Wang W., Liu X.M., Wang L., Sun Y. (2021). Mechanical behavior of outer square inner circular concrete filled dual steel tubular stub columns. Steel Compos. Struct..

[38] Farzampour A. (2017). Temperature and humidity effects on behavior of grouts. Adv. Concr. Constr..

